# The activity of phenoloxidase in haemolymph plasma is not a predictor of *Lymantria dispar* resistance to its baculovirus

**DOI:** 10.1371/journal.pone.0183940

**Published:** 2017-08-30

**Authors:** Nikita S. Kasianov, Irina A. Belousova, Sergey V. Pavlushin, Ivan M. Dubovskiy, John D. Podgwaite, Vyacheslav V. Martemyanov, Stanislav A. Bakhvalov

**Affiliations:** 1 Laboratory of ecological parasitology, Institute of Systematics and Ecology of Animals SB RAS, Novosibirsk, Russia; 2 Department of Natural science, Novosibirsk National Research State University, Novosibirsk, Russia; 3 Institute of Biology, Irkutsk State University, Irkutsk, Russia; 4 Laboratory of Insect Pathology, Institute of Systematics and Ecology of Animal SB RAS, Novosibirsk, Russia; 5 Novosibirsk State Agrarian University, Novosibirsk, Russia; 6 Northern Research Station, USDA Forest Service, Hamden, CT, United States of America; 7 Biological Institute, National Research Tomsk State University, Tomsk, Russia; Institute of Plant Physiology and Ecology Shanghai Institutes for Biological Sciences, CHINA

## Abstract

Host innate immunity is one of the factors that determines the resistance of insects to their entomopathogens. In the research reported here we studied whether or not phenoloxidase (PO), a key enzyme in the melanogenesis component of humoral immunity of insects, plays a role in the protection of *Lymantria dispar* larvae from infection by *L*. *dispar* multiple nucleopolyhedrovirus. We studied two types of viral infection: overt and covert. The following lines of investigation were tested: i) the intravital individual estimation of baseline PO activity in haemolymph plasma followed by virus challenging; ii) the specific inhibition of PO activity *in vivo* by peroral treatment of infected larvae with phenylthiourea (PTU), a competitive inhibitor of PO; iii) the evaluation of PO activity in the haemolymph plasma after larval starvation. Starvation is a stress that activates the covert infection to an overt form. All of these experiments did not show a relationship between PO activity in haemolymph plasma of *L*. *dispar* larvae and larval susceptibility to baculovirus. Moreover, starvation-induced activation of covert viral infection to an overt form occurred in 70 percent of virus-carrying larvae against the background of a dramatic increase of PO activity in haemolymph plasma in the insects studied. Our conclusion is that in *L*. *dispar* larvae PO activity is not a predictor of host resistance to baculovirus.

## Introduction

Gypsy moth *Lymantria dispar* L. (Lepidoptera: Eribidae) is a typical eruptive insect species that may cause defoliation over large areas. The range of *L*. *dispar* includes a large part of Eurasia (Western and Eastern Europe, Russia, and the Baltic states), countries of the Middle East and central Asia, northern China, Korea, North America (USA, Canada), as well as Japan and North Africa [1]. The *Lymantria dispar* multiple nucleopolyhedrovirus (LdMNPV) is a species-specific viral entomopathogen (family Baculoviridae), that may cause epizootics that impact host population dynamics. The virus has been developed for gypsy moth management [2,3]. Larvae become infected after peroral inoculation and die within 1–2 weeks as a result of mass reproduction of the virus in the host [4]. Larval cadavers are quickly liquefied by the action of chitinase, which is expressed at the terminal stage of pathogenesis [5]. Then the liquefied contents of the cadavers containing the occlusion bodies of virus are released into environment. Due to the protective chemistry of the occlusion bodies, gypsy moth LdMNPV can persist in the environment for long periods, perhaps years [6]. However, nucleopolyhedroviruses may persist asymptomatically in the host body and be activated by various stress factors [7–9]. Moreover, the virus can be transmitted from infected surviving parent to offspring through the egg [10–12]. This asymptomatic form is called the covert virus infection [13] and baculoviruses in this form of persistence are capable of replication [14, 13]. Currently, the role of covert baculovirus infection in the regulation of the host population density is still unclear [15].

Success of host infection by baculoviruses as well as by any other pathogens depends on insect’s immunity status, the pathogens potency, and the conditions of challenge. At present it is known that insect resistance to virus infection is determined by various physical and biochemical barriers: pH of midgut, properties of the peritrophic membrane, desquamation of epithelium, state of haemocytes (nodulation, encapsulation) and humoral immunity (signal path in cells, activity of phenoloxidase, antiviral peptides). Also, programmed cell death (apoptosis) and some regulatory intracellular processes like RNA interference (reviewed in [16]) can be involved.

Recently, during the study of the influence of environmental factors (phenology of the host plant) on susceptibility to LdMNPV we found negative association between phenoloxidase (PO) activity in insect’s haemolymph and the susceptibility of gypsy moth to LdMNPV infection [17]. This association was found for both types of infections, either overt or covert activated to overt [17]. It is known, that melanization during encapsulation and nodulation involves phenoloxidases (POs) which can hydroxylate tyrosine (enzyme EC 1.14.18.1) and also oxidize o-diphenols to quinones (enzyme EC 1.10.3.1). POs are copper-containing oxidoreductase enzymes, oxidizing phenolic compounds [18]. PO is found in insects as its inactive zymogen form, prophenoloxidase (proPO) [19]. The proPO is present in hemolymph (in plasma and hemocytes) and the integument [20]. In the integument, there is a third type of PO, laccase (enzyme EC 1.10.3.2.) [21]. Activation of proPO in insects occurs with the help of protease cascades, prophenoloxidase activating systems [22]. These proPO activating proteinases (PAPs) are present in the hemolymph as zymogens, and are activated in response to certain factors, including penetration by invaders [23–24]. PRRs (βGRP, PGRP, C-type lectins) bind to PAPs and this interaction leads to activation of initiator proteases which trigger a proteases cascade resulting in conversion of proenzyme PAPs to active proteinases [25]. Activated PAPs cleave proPO by limited proteolysis to form active PO [26]. PO is released from hemocytes by degranulation and deposited around wounds or encapsulated parasites. At the first stage in melanogenesis, hydroxylation of tyrosine to 3,4-dihydroxyphenylalanine (DOPA) occurs, then the oxidation of DOPA into DOPA-quinone [27–28]. There are a number of ROS (reactive oxygen species) and other intermediates linked with the melanotic cascade (o-quinones, hydrogen peroxide, o-semiquinone radicals etc.) that possess cytotoxicity and can destroy pathogenic microorganisms [27–29].

It has been shown that the phenoloxidase system plays an important function in encapsulation and nodulation reactions of invertebrate animals which are vital in defence against different parasites including bacteria, fungi, protozoa and parasitoids [30, 31]. For example, lowering defences via RNAi PPO easily led to infection of *Aeromonas hydrophila* by pathogenic bacteria [32]. When *Drosophila melanogaster* PPO1 and PPO2 (DmPPO1 and DmPPO2) were deleted, the mutants (PPO1Δ, PPO2Δ) were more susceptible to infection by gram-positive bacteria and fungi [33]. Also, insects use hindgut PPO to exterminate potential pathogens in the feces through melanization, thereby protecting their food from pollution by feces-transferred pathogens [34]. However, the role of PO in insect immunity against viral infection today is still being discussed. For example experiments with Indian mealmoth *Plodia interpunctella* and granuloviruses (*Baculoviridae*) have shown that PO activity does not affect host resistance to virus [35]. In the experiment with *Trichoplusia ni*, larvae which consumed cucumber leaves had higher activity PO in haemolymph compared with larvae eating cabbage leaves [36]. However, the larval susceptibility to baculovirus infection was independent of the host plant species. Moreover, time to death was faster in larvae feeding on cucumber leaves, that was assist with higher PO activity [36]. On the other hand it was shown that 5,6-dihydroxyindole is the melanogenesis intermediate which possess antiviral effects ex vivo [37]. McNeil and coauthors have shown the intensive accumulation of proPO during the antiviral response of gypsy moth larvae [38]. However, the activity of PO (activated from of proPO) was not significantly different compared with non-infected larvae [38]. Therefore, the role of PO activity in the insect resistance against baculoviruses is still speculative.

The aim of the current study is to test the hypothesis that phenoloxidase offers resistance to baculovirus infection in *Lymantria dispar*, an economically important species.

## Materials and methods

### Insects and virus strains

We used egg masses from a wild population of *L*. *dispar*, collected in the territory of Western Siberia in the autumn and held for winter diapause at + 4°C. Insects from these egg masses were used in the following spring after hatch. Also, a laboratory culture of *L*. *dispar*, initially originating from a New Jersey (NJ), USA gypsy moth population [39] was provided for our use by the USDA Forest Service. In order to induce the acute baculovirus infection we used strain *Ld*MNPV-27/0 (isolated in the territory of Western Siberia (55. 24^o^N, 75.82^o^E)) and strain *Ld*MNPV- NY isolated in the northeastern United States (NY) (42.70° N, 74.49° W) [40].

No permits for a field collection were required for this study, since the national forests in Russia were freely accessible. No protected species were sampled. Birch trees used for the feeding of insects in these studies were grown on the territory owned by the Russian Academy of Sciences.

### Experimental design

Baculoviruses posses the ability to persist outside of host and cause acute (overt) infection when the host ingests virus-contaminated food. These viruses also persist within the host organism (inducing covert infection) by mechanisms that as yet are not completely understood. We utilized both types of virus infection processes to test our hypothesis. We used several approaches in our research: i) the intravital individual estimation of base PO activity in haemolymph plasma followed by virus challenging (for overt infection only); ii) the specific inhibition of PO activity *in vivo* by peroral treatment with phenylthiourea (PTU), which is an competitive inhibitor of PO [41] (we used this approach for both covert and overt forms of infection); iii) the evaluation of PO activity in the haemolymph plasma following the influence of a stress factor (total starvation), which activates the covert infection to an overt form (we used this approach only for the covert form of infection). The evaluation of the prevalence of covert infections in *L*. *dispar* populations was performed by real-time PCR (see below).

### Intravital estimating of PO activity followed by exogenous virus infection

This work was conducted on the NJ laboratory culture of *L*. *dispar* larvae feeding on artificial diet. Haemolymph was collected from caterpillars 1 day after molting to the fourth instar. The PO activity was determined spectrophotometrically using L-DOPA as a substrate (2 mg/mL) and measuring the absorbance of reaction mixture at 490 nm. The detailed description of PO activity measured in *L*. *dispar* larval haemolymph is given in [42, 17]. In the current and following experiments the results of PO activity are represented in two ways: normalized to 1 μl of haemolymph plasma and normalized to 1 mg protein to control the protein concentration in the haemolymph plasma. To prevent the activation of proPO to PO induced by the cuticle puncture, 1mM soybean trypsin inhibitor (PMSF) was added to the haemolymph collection tube. Ten μl of haemolymph was collected from each larva following which insects were returned to feed on diet. A day later the punctured insects were used for virus infection. No deaths were recorded among larvae following puncturing and all punctured insects fed normally. The two virus strains used for the virus challenges (*Ld*MNPV-NY and *Ld*MNPV-27/0) were previously shown to differ significantly in potency [40]. This was done to compare the relationship between PO activity and the insect’s susceptibility to viral strains of varying potencies. Challenges were conducted using diet pieces treated with 5 μl of a viral occlusion body (OBs) suspension. The final virus dose was 10^5^ OBs on 1 piece. Due to the potency differences between strains we used 100 larvae for strain *Ld*MNPV-27 and 150 larvae for strain *Ld*MNPV-NY. For analysis of mortality we used only those insects that completely consumed contaminated diet pieces (79 larvae for *Ld*MNPV-27 and 133 larvae for *Ld*MNPV-NY). Thus all larvae were challenged with an identical virus dose. Mortality was recorded daily until pupation and the subsequent emergence of adults.

### *In vivo* inhibition of the activity of PO by PTU

The work was conducted on *L*. *dispar* larvae from a West Siberian population. Before using this approach we conducted a pilot experiment in which different concentrations of PTU were tested by peroral treatment to identify any toxic or antifeedant effects. A concentration of 0.1% did not lead to suppression of nutrition or to a loss of the mass of the tested larvae and this concentration was selected for further experimentation. The inhibition of larval PO activity was induced by spraying 10 ml of a 0.1% aqueous solution of PTU on the surface of silver birch (*Betula pendula* Roth.) leaves on a branch. There were 30 leaves, 5.02±0.22 cm in length and 4.46±0.30 cm in width, on each branch. Following treatment leaves were air-dried and 15 fourth-instar larvae were allowed to feed on leaves from a single branch. The activity of PO in the haemolymph plasma was determined one day after the larvae began to feed in accordance with methods described above. To avoid contamination by PTU during haemolymph collection, the haemolymph was taken from a puncture on the dorsal side of the larval body (not from the proleg as it is usually done). The activity of PO was determined as described above.

### Effect of PTU on the activation of covert infection

For this experiment, we used gypsy moth from a population with a high prevalence of covert LdMNPV infection (the method of LdMNPV detection is described below). Inhibition of phenoloxidase activity in the haemolymph was conducted by a single surface-spray on leaves with 0.1% PTU, as described above. The larvae were divided into two groups: 100 larvae treated with PTU and 100 control larvae feeding on leaves treated with water. Each group was divided into 5 replicates of 20 larvae. Each replicate of 20 larvae was reared in a 5 L plastic container. After consuming leaves, both PTU-treated and control larvae were fed native birch leaves washed in sterile distilled water to prevent potential microbial contamination. Mortality of larvae was recorded daily.

### Effect of PTU on the mortality of larvae induced by exogenous virus infection

The aim of this experiment was to test the exogenously induced LdMNPV pathogenesis under PTU inhibited PO activity. For this experiment we used the West Siberian population of *L*. *dispar* free from covert virus to exclude the interaction between the exogenous and endogenous viruses [9]. For the oral infection we used strain LdMNPV-27/0. We sprayed silver birch bouquets, with 10 ml of an aqueous suspension containing 2.4x10^7^ OBs/ml. This dose was given to 20 larvae. One hundred day-old fourth-instar larvae were used for the challenges. An additional one hundred larvae were fed leaves treated with distilled water and used as a control. Insects were reared in 5 L containers, 20 larvae/container as above. Five days after viral challenging, and following penetration of the midgut barrier (the peak LdMNPV-caused mortality is usually between 10 and 14 days) insects were peroraly treated with 0.1% PTU as described above. The protocol was to divide each group of control and infected larvae into 2 additional subgroups. One subgroup (i.e. 50 larvae) consumed leaves treated with PTU solution, while the other subgroup, consumed leaves treated with water. As a result, four groups of larvae were obtained: control, control+PTU, virus, virus+PTU. Cut birch branches were changed every other day and mortality was recorded until the adults emerged. Causes of larval mortality were confirmed by a light microscopy.

### Activation of a covert virus infection by the starvation

For this study, a population of gypsy moth larvae infected with a covert baculovirus was used. The level of the prevalence of the infection is shown in the results section. Total starvation was chosen as the stress factor to activate the covert form of infection to the overt form [43]. We starved larvae for four days starting the day after they molted to the fourth instar. We used two groups of larvae. One group was used for the daily recording of PO activity in the haemolymph plasma during the starvation (10 larvae/measurement according to the method described above). The other group (100 larvae) was used for recording the level of activation of the covert viral infection during the remaining ontogenesis. To exclude the starvation-induced cannibalism larvae were reared individually. Etiology of mortality was assessed by a light microscopy.

### Detection of covert baculovirus with PCR

In all cases, when working with wild populations, the covert viral infection was detected as follows. All eggs used for our experiments were cleaned of setae and surface sterilized by sodium hypochlorite to inactivate any LdMNPV on the surface of the eggs [44]. From each diapausing egg stock, 10 pools of 10 eggs in each (i.e. 100 eggs/stock) were randomly selected. If the studied population was already infected, 30 additional eggs from the pool were analyzed individually for clarifying the percentage of LdMNPV prevalence.

Total DNA was extracted using the phenol-chloroform method with some modification. The surface treated egg masses were mechanically homogenized using pestles in a lysis solution containing Guanidine thiocyanate. A control for successful DNA extraction was estimated using a PCR analysis of the 28S rRNA gene Lymantria dispar L [45].

A fragment of the polyhedrin gene was used as the target sequence for detecting the presence of LdMNPV DNA in the analysed samples. The amplification reactions for the analysis of the gypsy moth NPV polyhedrin gene contained primer1 50 nM, a primer2 50 nM, DNA 500 ng/25 μl at final volume, and the reaction mixture HS-qPCR Mix SYBR (Biolabmix). The reaction conditions were 5 min at 95°C, (30 sec at 95°C, 1 min at 60°C) for 40 cycles, with a melting curve of 60–95°C. The primers for LdMNPV polyhedrin gene were GCACTTCCTCAACTCGGTCA and CGTTTAGTACGCCGGTCCTT (Primer-BLAST). Viral DNA detection was performed by the using SYBR Green on CFX96 (Bio-Rad). It should be noted that the use of this qualitative detection method lowers the limit of DNA detection in comparison to standard detection by ethidium bromide. DNA extracted from the eggs of the gypsy moth population free of LdMNPV was used as the negative control. The product of the PCR reaction was sequenced to confirm the target sequence.

### Statistical analysis

The activity of PO was normally distributed in all samplings (for the estimation the Kolmogorov–Smirnov test was used) and a one-way ANOVA was used for the comparison of the data. Pearson linear correlation was performed to calculate the dependence between the PO activity of individual larvae assigned for infection and the duration of pathogenesis of the infected larvae. Larval mortality was compared by chi-square in the cases where no replicates were used. The mortality of larvae in the PTU study was compared by ANOVA. Prior to analysis, all data in percentages were the arcsine of the square root-transformed.

## Results

### Intravital estimating of PO activity and following exogenous challenge by LdMNPV

**Fig 1:**
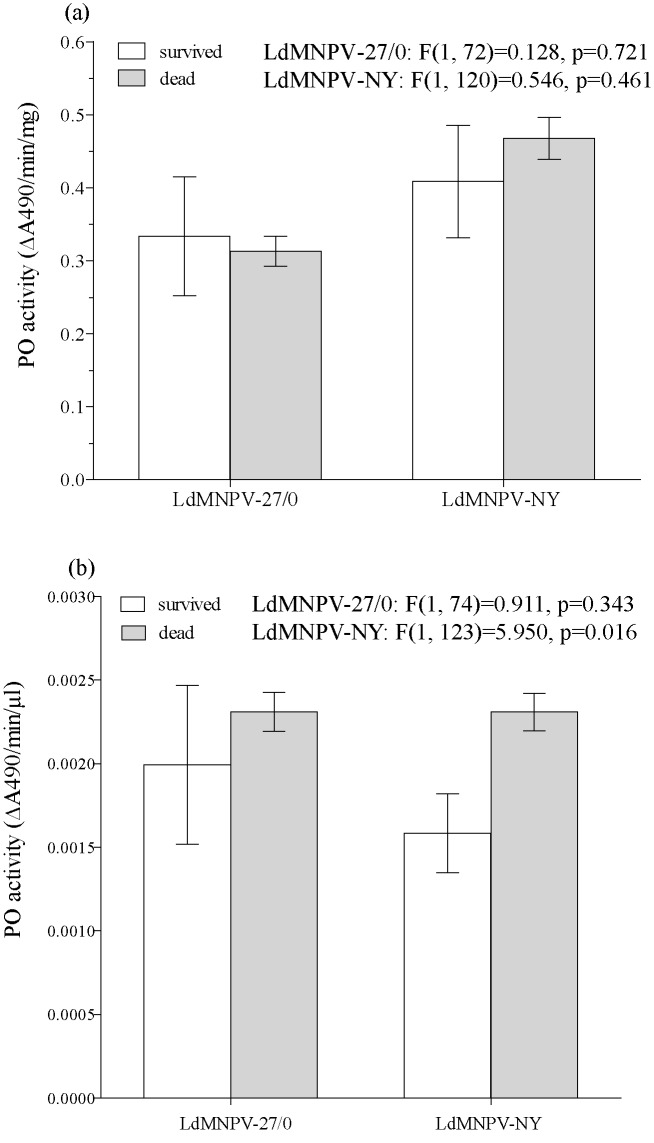
The comparison of PO activity between survived and dead after infection larvae of *Lymantria dispar*. PO activity was measured in haemolymph plasma of 4^th^ insar larvae. The challenging was done perorally with two different strains of LdMNPV: LdMNPV-27/0 isolated in Asia and LdMNPV-NY isolated in North America. PO activity is normalized to 1mg of protein (A) or to 1 μl of haemolymh (B). The results of the ANOVA comparision are given within the figure.

The total mortality rate for the group of larvae challenged by strain LdMNPV-27/0 was 80.8% while the mortality rate of insects challenged by strain LdMNPV-NY was higher—86,9% (χ^2^ = -2.23, p = 0.026). We did not find significant differences in PO activity of haemolymph plasma (recorded before the infection) between the pool of survivors and pool of dead larvae when insects were infected by the Siberian LdMNPV-27 strain (Fig 1A and 1B). The same result was shown for the infection by American LdMNPV-NY strain (Fig 1A), except when PO activity was normalized on the μl of haemolymph plasma. In that case dead after-infection-larvae showed higher basic PO activity (Fig 1B). We also analyzed the correlation between the basic PO activity and the speed of mortality after virus inoculation, assuming a positive correlation would result if the PO activity in plasma of haemolymph protected larvae from LdMNPV. No correlations were found: *r* = -0.210; p = 0.113 for larvae dead from the Siberian strain of virus; *r* = -0.174; p = 0.076 for larvae dead from the American strain of virus.

### The inhibiting of PO activity in plasma of haemolymph by PTU during LdMNPV infection

**Fig 2:**
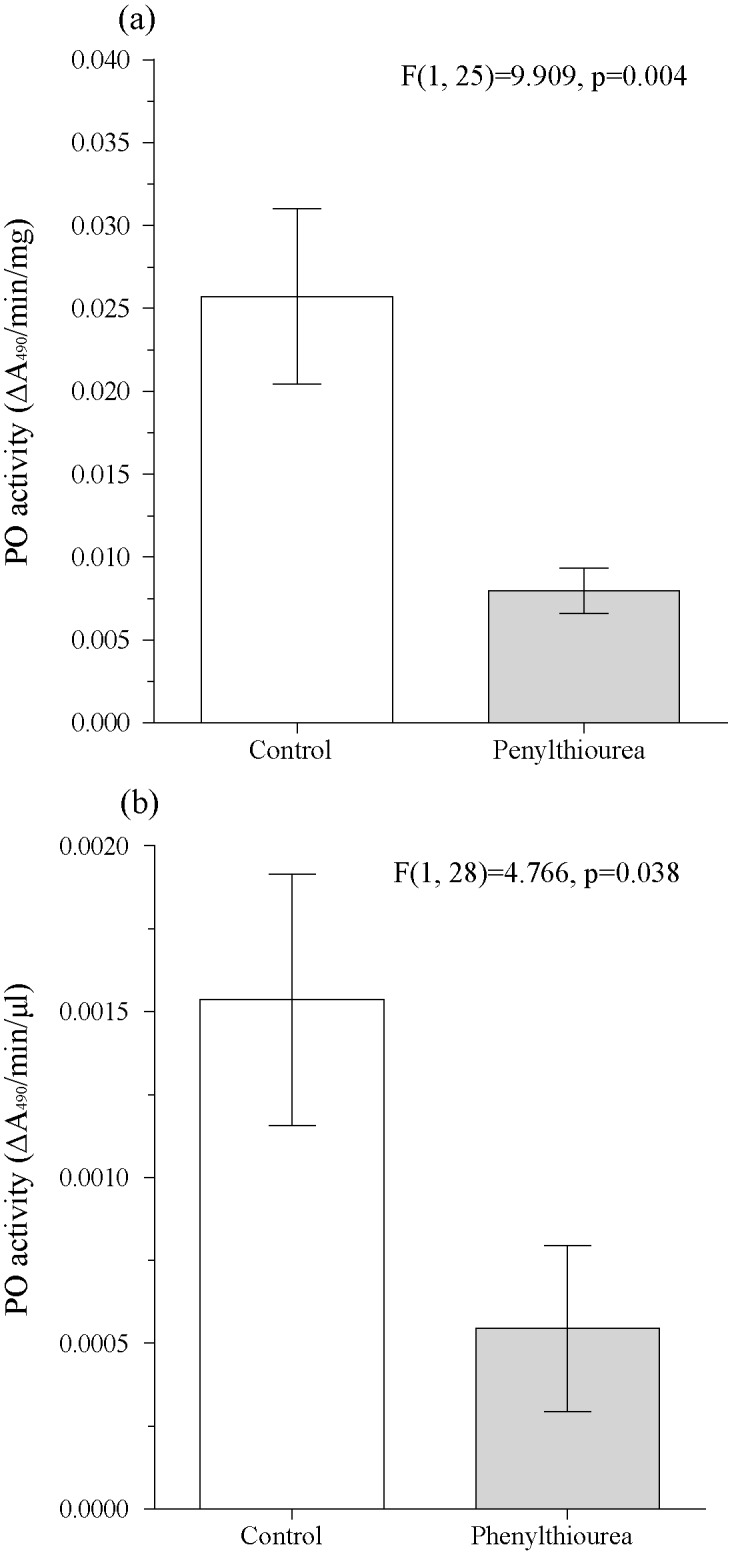
The effect of peroral PTU treatment on the *Lymantria dispar* larvae PO activity in haemolymph. PO activity measured in plasma of haemolymph of 4^th^ instar larvae was normalized to 1mg of protein (A) or to 1 μl of haemolymph (B). The results of the ANOVA comparision is given within the figure.

**Fig 3:**
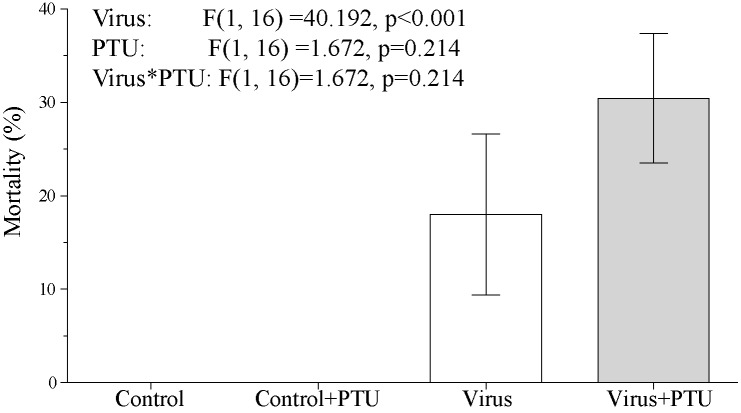
The effect of peroral PTU treatment on the LdMNPV induced mortality of *Lymantria dispar* larvae. The inoculation by virus was done perorally with the LdMNPV-27/0 strain. Real time PCR qualitative detection did not reveal that any eggs sampled from the stock were infected by covert virus. The results of the ANOVA comparision are given within the figure.

**Fig 4:**
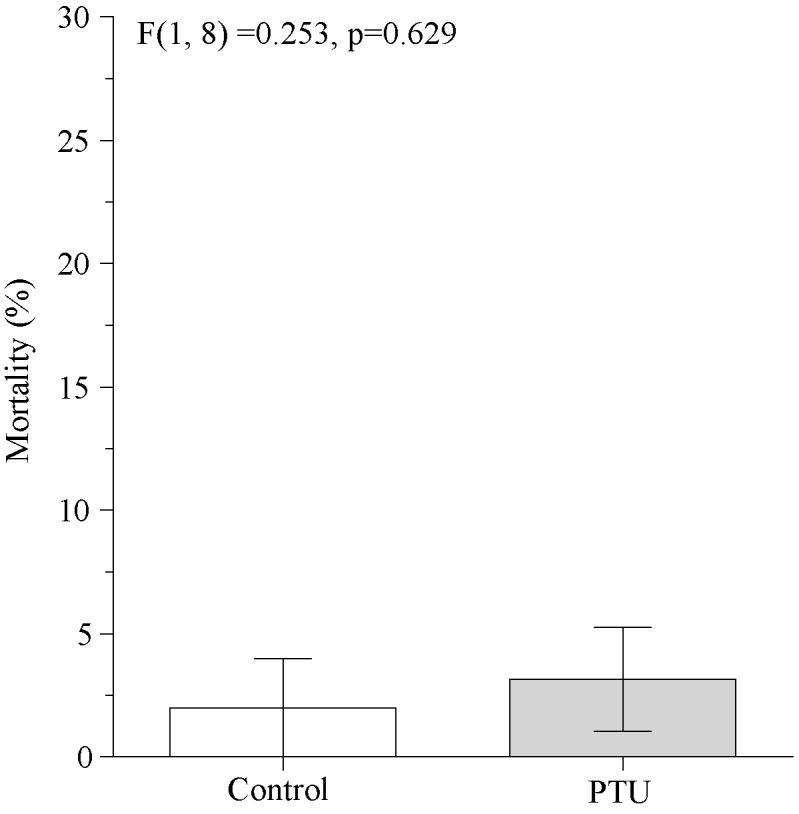
The effect of peroral PTU treatment on the LdMNPV-induced mortality of covertly infected *Lymantria dispar*. The prevalence of covert infection in *L*.*disapr* population, identified by real time PCR was 100%. The results of the ANOVA comparision are given within the figure.

Larvae feeding on leaves treated with 0.1% aqueous PTU showed a significant decrease of PO activity in the haemolymph plasma 1 day after treatment (Fig 2). This occurred independently of the rate of PO activation, the reaction parameter values, or the haemoymph protein concentration (Fig 2). We did not record a significant effect from the PTU peroral treatment on the mortality rate of larvae exogenously challenged by LdMNPV while the virus alone significantly induced virus-specific mortality (Fig 3). We also did not find an increase in the activation of covert baculovirus to overt infections in insect groups that were highly covertly-infected when they were perorally treated with PTU (Fig 4).

### The estimating of PO activity in plasma of haemolymph during activation of covert form of virus to overt form by starvation

**Fig 5:**
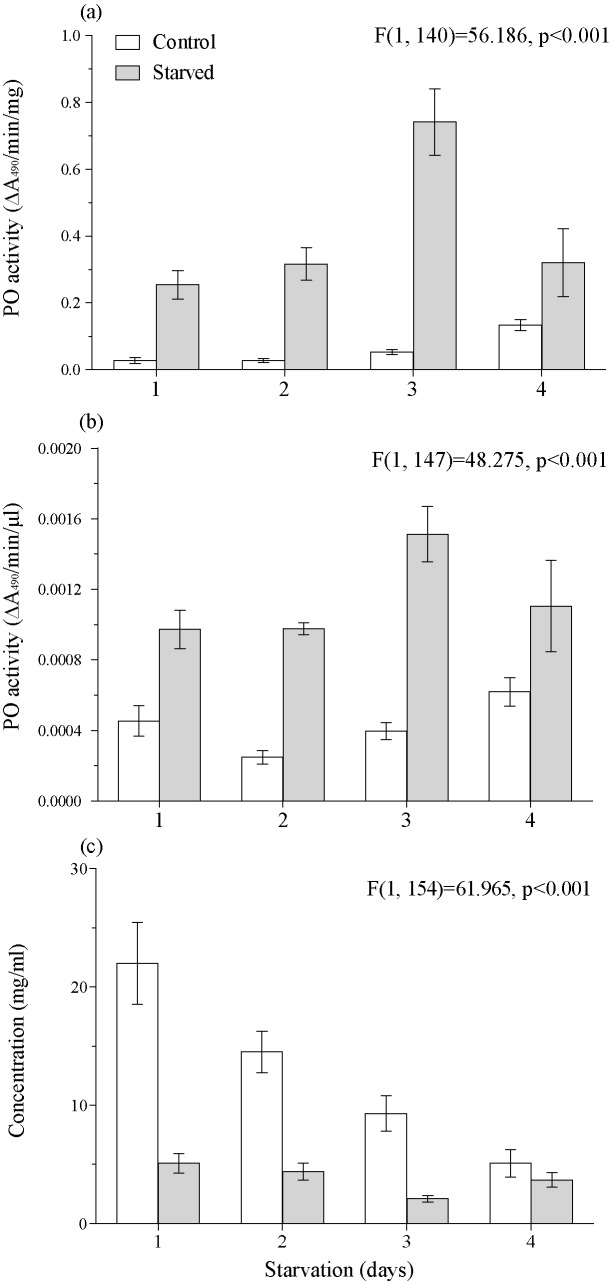
The effect of starvation on the PO activity in heamolymph of *Lymantria dispar* larvae. PO activity measured in plasma of haemolymph of 4^th^ instar larvae was normalized to 1mg of protein (A) or to 1 μl of haemolymh (B). The concentration of protein in plasma of haemolymph (C). The results of the ANOVA comparision are given within the figure.

**Fig 6:**
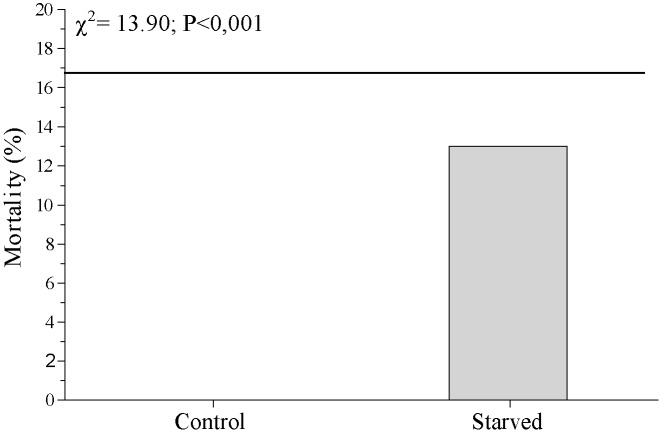
The LdMNPV induced mortality of *Lymantria dispar* larvae starved during four days. The line indicates the prevalence of covert LdMNPV in the studied *L*. *dispar* population. The statistical results of the comparison between starved and control larvae are given within the figure.

The starvation of larvae led to significant increases of PO activity from the first day of the treatment. These increases in PO activity were not determined by the decrease of protein concentration in haemolymph plasma because of a total restriction in food intake (Fig 5). On the other hand, in larvae that were starved for 4 days we recorded a 13% activation level of LdMNPV-induced mortality in the population that carried covert virus (Fig 6). The PCR detection revealed that the prevalence of covert LdMNPV was 17% (Fig 6). Thus we registered around a 70% virus activation level under the starvation treatment.

## Discussion

In many insect species the functioning of PO in haemolymph determines the host resistance to parasites [46–48], parasitoids [49–52] and some microorganism [51–52]. However this is not the case for the resistance to representatives of the Baculoviridae family and there are several contradicting studies showing this. For example, the experiments of Saejeng and co-authors [35], using granuloviruses and *Plodia interpunctella*, showed that the activity of PO in haemolymph plasma of both infected and uninfected larvae did not differ. Offspring of nutritionally stressed Indian meal moths, *Plodia interpunctella*, had heightened resistance to a baculovirus [53], while another study demonstrated reduced immune activity (i.e. haemocyte density and phenoloxidase activity) in the same insect species [54]. Shikano and co-authors [36] did not find an association of PO activity in the haemolymph plasma with the resistance of larvae *Trichoplusia ni* to *Trichoplusia ni* single nucleopolyhedrovirus when exposed to infection under different quality of nutrition of the parent and daughter generations of insects. On the other hand, Zhao and coauthors [37] showed that the melanin precursor (5,6-dihydroxyindole), had a direct antiviral effect against baculovirus. However, this effect was showed *ex vivo*. The negative effect of the reactive oxygen species (ROS) formed by melanogenesis on viruses has also been shown *in vitro* in the experiment with *Heliothis virescens* [55]. Although, these reactive compounds do have virucidal properties, this effect could not easily be extrapolated on the whole host organism. This restriction is due to the fact that insects, like many other multicellular organisms, have antioxidant protection system, which function to prevent nonspecific damaging of biopolimers by ROS. In particular, the formation of ROS in insects is controlled by enzymatic processes (superoxidedismutase, catalase, ascorbate enzymes and glutathione-dependent cycles) and non-enzymatic antioxidants such as thiol- and phenol-containing compounds (reviewed in [56, 57]). *L*. *dispar* used in our study also has antioxidant mechanisms including: superoxide dismutase, catalase, glutathione-S-transferase, glutathione reductase [58, 59]. Therefore, the functioning of antioxidant systems in insects may significantly modify (decrease) the virucidal ability of reactive molecules produced during melanogenesis observed *in vitro* [55, 37]. There is an elegant study where researchers used several molecular approaches to demonstrate that PO in the U4.4 cell line from *Aedes albopictus* possessed antiviral activity against Semliki Forest Virus (Togaviridae); this effect was reproduced at the organism level [60]. However, baculoviruses are highly complex, organized viruses compared to the simple RNA-containing Semliki Forest Virus. In Particular, buculoviruses have many genes with still poorly described functions [5]. Some of these genes are homologues to the superoxide dismutase gene [5], whose protein is an antioxidant enzyme catalyzing the transformation of a superoxide anion to hydrogen peroxide. A recent study has confirmed the important role of this virus gene for virus propagation [61]. Thus, differently organized viruses may use significantly different strategies for host–pathogen interactions and additional studies are needed to confirm antiviral functioning of PO *in vivo* for a variety of species [55, 37].

All approaches used in current study showed the absence of a relationship between the susceptibility *L*. *dispar* to baculovirus and the PO activity in haemolymph plasma of *L*. *dispar* larvae. This was shown for both covert and overt types of viral infections. In particular, the larvae under suppression of PO with a specific inhibitor did not show enhanced susceptibility to virus. We did not find a correlation between intravital PO activity in larvae and the speed of viral pathogenesis. Moreover the induction of PO in larvae by starvation did not enhance protection against virus infection. In the investigation of the development larval resistance to exogenous baculovirus challenge, a significant increase of proPO three days after infection by LdMNPV was detected [38]. Whereas the activity of the activated enzyme (PO) has been shown to remain at the same level independent of infection [38]. The high activity of the zymogen is speculated by the authors only as a potential defence by the proPO cascade against baculovirus, although based on the results shown in Fig 3A of McNeil co-authors [38], it seems that this potential could not be realized during pathogenesis. Thus the role of elevated proPO in the haemolymph of *L*. *dispar* larvae induced by LdMNPV infection is still unexplained and needs to be studied further.

We expected that if PO activity was related to host resistance against baculovirus it would have been detected in the study of covert infection vertically transmitted from the parent. In that case virus has already overcome midgut barrier defence and gut based resistance would not effect the relationship between PO activity and resistance to virus. However, in our PO inhibition experiment our results showed that even when the virus was already covertly located inside the host organism no negative relationship between PO activity and cases of virus activation was found. The same result was shown in our study on the activation of covert infection by starvation, where starved virus-carrying larvae were dead in the 70% of cases against the background of significant increasing PO activity in haemolymph plasma. In other insect species long-term starvation leads to a decrease in immunity functions [62, 63], while short-term starvation only leads to a reversible decrease in the activity of the immune system, which is restored upon return to normal feeding regimen [62]. That is not in agreement with data obtained in our experiments but partially in agreement with results obtained by [64], where a significant increase of PO activity was shown during the starvation of *Epirrita autumnata*. The functioning of PO in insects is directly dependent on the amount of consumed protein [65], so it is difficult to explain the increased PO activity during the starvation (i.e. total absence of consumed protein) observed in our study and in the study of Yang and coauthors [64]. Possibly it is the consequence of a trade-off between the functioning of the proPO cascade and other immune parameters. Whatever the reasons for the activation of PO by *L*. *dispar* starvation it is obvious that the functioning of this enzyme does not prevent the development of virus pathogenesis, which is a transformation from covert infection to overt infection during starvation.

Thus, using several lines of evidences we showed that the activity of PO in haemolymph plasma of *L*. *dispar* larvae dose not predict host susceptibility to baculovirus. Our findings indicate that the decrease of PO activity and the increase of *L*. *dispar* larvae susceptibility to baculovirus under the effect of host plant–herbivore developmental asynchrony [17] are not directly linked and that an asynchronous mediated increase of larval susceptibility to baculovirus is related with another (still unstudied) immune parameter possibly covariated with PO activity. Further studies are needed to determine the role of host immune function in host resistance to baculovirus infection, in particular the covert form of infection.

## Supporting information

S1 Dataset Initial dataset of all experiments described within the article. (XLS)
